# Hostility and violence of acute psychiatric inpatients

**DOI:** 10.1186/1745-0179-1-11

**Published:** 2005-07-29

**Authors:** Michele Raja, Antonella Azzoni

**Affiliations:** 1 Università degli Studi di Roma "La Sapienza". Scuola di Specializzazione in Psichiatria. Servizio Psichiatrico di Diagnosi e Cura, Ospedale Santo Spirito, Rome, Italy

**Keywords:** hostility, violence, psychiatry, emergency, psychosis, schizophrenia, mood, disorders

## Abstract

**Objective:**

The aim of the present study was to find out the extent of hostility and violence and the factors that are associated with such hostility and violence in a psychiatric intensive care unit.

**Methods:**

Retrospective analysis of data prospectively collected in a 6-year period.

**Results:**

No hostility was observed in 56.1%, hostility in 40.9%, and violence in 3.0% of the admitted cases. Seclusion was never used. Six cases (2,5‰) required physical restraint. Risk factors associated with violence were younger age, suicidal risk, and diagnosis of schizophrenia. Risk factors associated with hostile and violent behavior were younger age at the onset of the disorder, being single, having no children, lower GAF scores, higher BPRS hostility, SAPS, and CGI scores, lower BPRS anxiety-depression score, higher doses of psychoactive drugs, more frequent use of neuroleptics, diagnosis of mania, personality disorder, substance and alcohol related disorders, no diagnosis of depression.

**Conclusion:**

The study confirms the low rate of violence among Italian psychiatric in-patients, the major relevance of clinical rather than socio-demographic factors in respect of aggressive behavior, the possibility of a no seclusion-no physical restraint policy, not associated either with higher rates of hostility or violence or with more severe drug side effects.

## Introduction

Hostility and violence have long been a matter of concern in inpatient psychiatry. Violence of inpatient psychiatric units is a distinct character from outpatient violence. Of inpatients, 18% to 25% exhibit violent behavior while in the hospital [1,2]. Of violent acts, 78% are directed to nurses, with other targets being (in descending order of frequency) fellow patients, property, self, physicians, psychologists, family members, and housekeeping staff [3]. Ten to 45% of patients with schizophrenia exhibit aggressive or threatening behavior during hospitalization [4-7]. Since violence is a complex behavior related to clinical as well as social components and approaches of psychiatric care, it is particularly important to investigate the aggressive and violent behavior of psychiatric patients in different settings and countries in order to find out risky or protective factors. In Italy, reported rates of psychiatric inpatients' violent behavior tend to be lower than in other countries [8-10]. The reasons are unknown. The aim of the present study was to find out the extent of hostility and violence and the factors that are associated with such hostility and violence in a general hospital Psychiatric Intensive Care Unit (PICU).

## Methods

The study was carried out at a12 bed PICU of a general hospital with a catchment area of about 210.000 inhabitants. In this area, most voluntary patients and all involuntary patients who need psychiatric hospitalization are admitted to this PICU. Some milder cases are admitted to private clinics. Admissions exclude persons under age 18. As the hospital is in the center of Rome, near St. Peter's Basilica, we also accept foreign patients with different backgrounds. We do not think our population of patients to be unique if compared to psychiatric patients in general. Ten psychiatrists, one psychologist, one social worker and 15–20 nurses work in the ward. In the years 1997–98, 1999–2000, and 2001–2002, the PICU was allocated in 3 different buildings, each with peculiar architectonic features. The patients examined were all those discharged between 1 January 1997 and 31 December 2002. The following data were ascertained for each patient: sex, age, diagnosis, type of admission (voluntary or involuntary), length of hospitalization, psychopharmacological treatment on admission and on discharge. A chlorpromazine equivalent dose of antipsychotics [11] and a diazepam equivalent dose of benzodiazepines [12] were considered. One hundred mg of chlorpromazine or 2,5 mg of haloperidol were considered equivalent to 1,6 mg of risperidone, 5 mg of olanzapine, 200 mg of quetiapine, 50 mg of clozapine, 4 mg of sertindole. We used a modified version of the Morrison's scale [13] to rate patients' highest level of hostile or violent behavior during hospitalization. The anchor points of the modified version used in the study are the following: 0: "no hostility"; 1: "exhibited low-grade-hostility"; 2: "was loud and demanding", 3: "approached another in a threatening way"; 4: "made a verbal threat without a plan to inflict a harm"; 5: "is violent against objects"; 6: "touched another in a threatening way"; 7: "made a verbal threat with a plan to inflict a harm"; 8: "inflicted low-grade harm requiring no medical care"; 9: "inflicted serious harm requiring medical care". For purposes of data analysis, the nine levels rated by the scale were combined into three classes of increasingly severe aggressive behavior: a) *no hostility *(score 0); b) *hostility *(scores 1–7); c) *violence *(scores 8–9). In as many patients as possible, as part of clinical routine, we registered years of education, social class, age at the onset of the disorder, and assessed on admission clinical conditions by the Brief Psychiatric Rating Scale (BPRS), including 24 items rated from 1 to 7 [14], the Scale for the Assessment of Positive Symptoms (SAPS) [15], the Scale for the Assessment of Negative Symptoms (SANS) [16], the Mini Mental State Examination (MMSE) [17], the Global Assessment of Functioning Scale (GAF) [18] and the Clinical Global Impression (CGI). The duration of the time frame for assessment was 7 days for the BPRS, SAPS and SANS. Social class was rated using an original scale that considers the years of education and the employment status of the patient and of the head of his/her family, and the residence of the patient [1-5] point scoring system for each item, range of total score: 5–25). Suicidal risk was assessed by a questionnaire including 5 yes/no answers. At least two yes answers were considered to be necessary to define the suicidal risk present. For purposes of data analysis, we combined the BPRS symptom scales into four summary scores: 1) *Psychotic cluster *which includes *Conceptual disorganization*, *Grandiosity*, *Hallucinatory behavior*, and *Unusual thought content*; 2) *Withdrawal-Retardation cluster *which includes *Motor retardation*, *Emotional withdrawal*, and *Blunted affect; 3) Hostility-Suspiciousness cluster *which includes *Hostility*, *Suspiciousness*, and *Uncooperativeness; 4) Anxiety-Depression cluster *which includes *Anxiety*, *Depression*, and *Guilt*. Neurological examination included the use of the Abnormal Involuntary Movement Scale (AIMS) [19], the Unified Parkinson's Disease Rating Scale (UPDRS) [20], and the Barnes Akathisia Scale (BAS) [21]. No distinction was made in the analyses between alcohol or drug abuse and dependence. The χ_2 _test was used to analyze categorical variables. T-test (comparison between two groups) and analysis of variance with Bonferroni test (comparison between three groups) were performed for continuous variables. All p values were two tailed, and statistical significance was set at p < 0.05.

## Results

In the considered period, 2395 cases, 1067 men (44.6%) and 1328 women (55.4%) were admitted to the PICU. Involuntary admissions were 604 (25.2%). Patients' mean age was 41.9 (± 14.1) years, mean years of education were 10.7 (± 3.9), and mean social class score was 14.2 (± 4.3). Of the admitted cases, 1331 were single, 449 married, 184 separated, 97 divorced, 104 widows or widowers (civil status not determined in 230 cases), 638 had children, 1100 had no children (parenthood not determined in 657 cases). Ethnic background was caucasic in 98% of cases. The most frequent diagnoses were schizophrenia (295, 12.3%), schizoaffective disorder (348, 14.5%), bipolar disorder *mania *(386, 16.1%), *depression *(99, 4.1%), *mixed episode *(322, 13.4%), unipolar depression (113, 4.7%), dysthymic disorder or depression NOS (53, 2.2%), psychotic disorder NOS (379, 15.8%), delusional disorder (23, 1.0%), obsessive-compulsive disorder (OCD) (21, 0.9%), dissociative disorders (29, 1.2%), alcohol or substance related disorder (78, 3.3%), personality disorder (55, 2.3%), behavioral misconduct related with mental retardation (83, 3.5%) or with dementia (17, 0.7%), delirium, mood or psychotic disorder due to general medical condition (19, 0.8%), Asperger's disorder (9, 0.4%), eating disorders (7, 0.3%). Non-hostile cases (Morrison score of 0) were 1322 (56.1%), hostile cases (Morrison score 1–7) were 962 (40.9%), and violent cases (Morrison score of 8–9) were 70 (3.0%) (Morrison score not reported in 41 cases). Their demographic and clinical characteristics are summarized in the Tables 1, 2, 3, 4. Hostility or violence were directed against self in 12 (1.2%) cases, other patients in 75 (7.3%) cases, patients' relatives in 73 (7.1%) cases, visitors in 4 (0.4%) cases, staff in 933 (90.4%) cases, objects in 38 (3.7%) cases. No patient was moved from the PICU to intensive medical care units because of treatment-related side effects. No fatality occurred. Two mild cases of neuroleptic malignant syndrome were observed. Both of them rapidly resolved after neuroleptic discontinuation and medical support. Seclusion was never used. Six cases (2,5‰) required physical restraint (PHR), two of them for more than one day. PHR was used because of medical illness that contraindicated the use of psychoactive drugs or in the presence of persistent violent behavior in spite of the use of high doses of psychoactive drugs. Most assaults were not a significant threat to the attacked person, but few were highly dangerous. Violent cases were younger in comparison with the other two groups. Violent cases were more likely to be single and to have no children than hostile cases. The latter were more likely to be single and to have no children than non-hostile cases. Hospitalization was longer in violent than in hostile cases and in hostile than in non-hostile cases. The interval between admission and the complete neuropsychiatric assessment was longer in hostile and violent than in non-hostile cases. There was no difference among the three groups in terms of years of education and social class. Commitment was more frequent in hostile than in non-hostile cases, and in violent than in hostile cases. Current and last year best GAF scores were lower in violent than in hostile cases and in hostile than in non-hostile cases. Non-hostile cases were older than the other two groups at the onset of their psychiatric disorder. BPRS psychotic cluster score was higher in hostile than in non-hostile cases. BPRS hostility and SAPS scores were higher in hostile and violent cases than in non-hostile cases. BPRS anxiety-depression score was lower in hostile and violent cases than in non-hostile cases. SANS score was higher in violent and in non-hostile than in hostile cases. UPDRS rigidity and akinesia scores were higher in non-hostile than in hostile cases. Regarding drug treatment (see Tables 3 and 4), antipsychotic and benzodiazepine daily doses were higher in violent than in hostile cases and in hostile cases than in non-hostile cases, both on admission and discharge. On admission, VPA daily dose was higher in violent than in hostile cases and in hostile cases than in non-hostile cases. On discharge, VPA daily dose was higher in violent and in hostile cases than in non-hostile cases. Typical neuroleptics were more frequently used in hostile and in violent cases than in non-hostile cases. CGI score was higher in violent than in hostile cases and in hostile than in non-hostile cases. Suicidal risk was higher in the violent than in the other two groups. Regarding diagnosis, schizophrenia was more frequent in the violent than in the other two groups, mania was more frequent in the hostile and violent groups than in the non-hostile group, while the opposite was true for depressive states, more frequent in the non-hostile than in the other two groups. Personality disorders, substance and alcohol related disorders were more frequent in the violent and in the hostile than in the non-hostile group. There was no difference in the rate of hostility or violence among the 6 considered years. The rates of hostility and violence were similar in the three consecutive architectural settings of the PICU.

**Table 1:** Sex, commitment, suicidal risk, and diagnosis in the non-hostile, hostile and violent groups

	No hostility	Hostility	Violence	X2	df	P
**Cases**	1322 (56.1%)	962 (40.9%)	70 (3.0%)			
**Men/Women**	581 / 741	433 / 529	31 / 39	0.255	2	.880
**Voluntary/involuntary**	1175 / 143	559 / 401	30 / 40	324.412	2	.000*
**Civil status**						
Single	693 (58.6%)	560 (63.3%)	56 (83.5%)	19.148	2	.000*
Married	282 (23.9%)	162 (18.3%)	3 (4.5%)	20.684	2	.000*
Separated	91 (7.7%)	84 (9.5%)	4 (6.0%)	2.667	2	.263
Divorced	56 (4.7%)	35 (4.0%)	3 (4.5%)	0.728	2	.695
Widow/widower	60 (5.1%)	43 (4.9%)	1 (1.5%)	1.756	2	.416
Parenthood	372 (39.6%)	246(34.4%)	9 (15.0%)	17.234	2	.000*
**Suicidal risk**	288 (42.0%)	149 (36.9%)	32 (71.1%)	53.926	2	.000*
**Diagnosis**						
Schizophrenia	177 (13.4%)	93 (9.7%)	16 (22.9%)	14.975	2	.000*
Schizoaffective disorder	182 ((13.8%)	155 (16.1%)	10 (14.3%)	2.449	2	.294
Unipolar depression	97 (7.3%)	25 (2.6%)	1 (1.4%)	27.348	2	.000*
Bipolar depression	82 (6.2%)	16 (1.7%)	1 (1.4%)	29.862	2	.000*
Depression NOS	41 (3.1%)	6 (0.6%)	0	18.939	2	.000*
Mania	133 (10.1%)	234 (24.3%)	11 (15.7%)	84.052	2	.000*
Bipolar mixed state	162 (12.3%)	148 (15.4%)	9 (12.9%)	4.688	2	.096
Psychotic disorder NOS	225 (17.0%)	130 (13.5%)	9 (12.9%)	5.611	2	.06
Personality disorder	66 (5.0%)	92 (10.0%)	12 (17.1%)	27.962	2	.000*
Alcohol related disorder	113 (8.5%)	119 (12.4%)	9 (12.9%)	9.391	2	.009*
Substance related disorder	81 (6.1%)	102 (10.6%)	13 (18.6%)	24.535	2	.000*
Tardive dyskinesia	127 (30.8%)	81 (32.3%)	8 (25%)	3.915	2	.141

* = statistically significant

**Table 2:** Age, length of hospitalization, time of assessment, educational level, social class, clinical variables in the non-hostile, hostile and violent groups

	No hostility	Hostility	Violence	Variance analysis	Df	Bonferroni test; p < .05
Age (years)	42.2 (± 13.8)	41.9 (± 14.4)	36.3 (± 12.3)	F = 6.02 P = .002	2341	1 vs 3 = yes 2 vs 3 = yes 1 vs 2 = no
Hospitalization (days)	9.7 (± 12.5)	12.8 (± 13.8)	20.7 (± 17.4)	F = 32.99 P = .000	2348	1 vs 3 = yes 2 vs 3 = yes 1 vs 2 = yes
Interval admission/complete assessment (days)	3.0 (± 4.6)	4.0 (± 6.0)	5.8 (± 7.5)	F = 9.73 P = .000	1279	3 vs 1 = yes 2 vs 1 = yes 3 vs 2 = no
Education level (years)	10.5 (± 4.0)	10.9 (± 3.8)	10.7 (± 3.4)	F = 1.52 P = .220	1370	NS
Social class	14.3 (± 4.2)	14.2 (± 4.4)	13.6 (± 3.7)	F = 0.6 P = .546	1353	NS
GAF (current score)	24.6 (± 7.9)	23.3 (± 6.9)	19.8 (± 7.6)	F = 12.24 P = .000	1339	1 vs 3 = yes 1 vs 2 = yes 2 vs 3 = yes
GAF (best score in the last year)	49.6 (± 14.5)	47.2 (± 13.7)	41.1 (± 13.3)	F = 11.06 P = .000	1296	1 vs 3 = yes 1 vs 2 = yes 2 vs 3 = yes
Age at the beginning of illness (years)	29.4 (± 13.7)	27.0 (± 12.8)	21.9 (± 8.8)	F = 6.84 P = .001	933	1 vs 3 = yes 1 vs 2 = yes 2 vs 3 = no
BPRS total	57.0 (± 13.2)	61.4 (± 13.3)	61.5 (± 13.6 =	F = 17.49 P = .000	1278	NS
BPRS psychotic cluster	10.2 (± 5.1)	11.6 (± 5.1)	11.2 (± 4.8)	F = 11.85 P = .000	1278	2 vs 1 = yes 2 vs 3 = no 3 vs 1 = no
BPRS withdrawal/retardation	7.6 (± 4.4)	6.2 (± 3.9)	7.6 (± 4.3)	F = 15.96 P = .000	1278	NS
BPRS hostility/agitation	6.6 (± 3.0)	9.7 (± 3.6)	9.9 (± 4.4)	F = 138.93 P = .000	1278	3 vs 1 = yes 2 vs 1 = yes 3 vs 2 = no
BPRS anxiety/depression	9.9 (± 4.5)	8.1 (± 4.1)	7.6 (± 4.0)	F = 28.60 P = .000	1278	1 vs 3 = yes 1 vs 2 = yes 2 vs 3 = no
SAPS	33.1 (± 23.6)	41.3 (± 22.1)	42.1 (± 20.4)	F = 20.83 P = .000	1278	3 vs 1 = yes 2 vs 1 = yes 3 vs 2 = no
SANS	48.4 (± 24.7)	43.6 (± 23.6)	55.1 (± 21.6)	F = 8.99 P = .000	1279	2 vs 3 = yes 2 vs 1 = yes 3 vs 1 = no
MMSE	26.5 (± 3.3)	26.4 (± 2.9)	26.0 (± 4.3)	F = 0.59 P = .557	1231	NS
UPDRS total	7.1 (± 6.4)	6.0 (± 5.5)	7.4 (± 8.2)	F = 5.19 P = .557	1206	NS
UPDRS rigidity	0.5 (± 0.7)	0.4 (± 0.6)	0.5 (± 0.7)	F = 3.77 P = .023	1206	1 vs 2 = yes 1 vs 3 = no 3 vs 2 = no
UPDRS tremor	1.5 (± 1.7)	1.2 (± 1.5)	1.6 (± 1.6)	F = 4.43 P = .012	1206	NS
UPDRS akinesia	0.9 (± 1.2)	0.7 (± 1.0)	0.7 (± 1.3)	F = 4.25 P = .015	1204	1 vs 2 = yes 1 vs 3 = no 3 vs 2 = no
Barnes akathisia scale	0.5 (± 1.0)	0.4 (± 0.9)	0.5 (± 1.0)	F = 1.52 P = .220	1203	NS

**Table 3:** Psychoactive drug doses used in the treatment of the non-hostile, hostile and violent groups

Treatment	No hostility	Hostility	Violence	Variance analysis	df	Bonferroni test; p < .05
CPZ Admission dose (mg)	313.55 (± 252.7)	386.44 (± 364.7)	645.05 (± 713.3)	F = 21.89 P = .000	1051	3 vs 1 = yes 3 vs 2 = yes 2 vs 1 = yes
CPZ Discharge dose (mg)	390.58 (± 329.8)	495.82 (± 432.4)	765.0 (± 585.12)	F = 33.55 P = .000	1637	3 vs 1 = yes 3 vs 2 = yes 2 vs 1 = yes
DZ Admission dose (mg)	19.2 (± 14.4)	28.4 (± 20.8)	39.5 (± 32.4)	F = 29.71 P = .000	636	3 vs 1 = yes 3 vs 2 = yes 2 vs 1 = yes
DZ Discharge dose (mg)	18.5 (± 16.1)	29.9 (± 22.9)	39.3 (± 30.5)	F = 37.51 P = .000	738	3 vs 1 = yes 3 vs 2 = yes 2 vs 1 = yes
LI Admission dose (mg)	715.0(± 316.6)	728.8(± 297.9)	835.7(± 170.1)	F = 0.53 P = .591	222	NS
LI Discharge dose (mg)	852.4(± 237.1)	856.8(± 272.4)	930.0(± 290.2)	F = 0.64 P = .530	384	NS
VPA Admission dose (mg)	702.4(± 309.1)	847.0(± 356.6)	1094.0(± 364.8)	F = 20.17 P = .000	554	3 vs 1 = yes 3 vs 2 = yes 2 vs 1 = yes
VPA Discharge dose (mg)	894.4(± 677.0)	976.4(± 381.2)	1184.6(± 350.6)	F = 8.82 P = .000	869	3 vs 1 = yes 2 vs 1 = yes 3 vs 2 = no

CPZ = chlorpromazine equivalent, DZ = diazepam equivalent, LI = lithium, VPA = valproate

**Table 4:** Use of antidepressants, typical, atypical, and depot antipsychotics in the treatment of the non-hostile, hostile and violent groups

Treatment	No hostility	Hostility	Violence	X2	df	P
Antidepressants	167	42	3	48.393	2	.000*
Atypical antipsychotics/Typical antipsychotics	665/342	542/347	43/37	7.285	2	0.026*
Only atypical antipsychotics/Only typical antipsychotics	570/247	438/243	29/23	9.345	2	.009*
Depot yes/no	137/864	150/702	18/44	13.499	2	.001*

## Discussion

The strengths of the study include: 1) The observation of a large series of unselected acute psychiatric in-patients who were well characterized clinically. 2) The risk of underreporting violence seems to be low because the data about patients' violence were collected prospectively considering several sources of information such as medical and nurses' records, daily meetings of staff members, and patients' and family members' reports. There are also several weaknesses that should be noted: 1) The study was carried out at a single facility. Specific hospital practices and regional characteristics may have influenced the results. Studies carried out in other institutions may be helpful, but there are so many differences among settings that an examination of each hospital's unique pattern of violence is necessary. 2) Data were collected systematically and uniformly and without the purpose of being used for specific research. The use of such data may reduce the risk of evaluation bias. However, the selection of variables that can be included in the analysis is dependent on the availability of data in registers, making some variables of interest, e.g. the number and the type of assaults made by each patient, the hour and the place of assaults, the context of the violent episode, absent in this study. 3) The distinction between primary from secondary diagnoses can sometimes be difficult, if not impossible. Therefore, in the analysis, we considered together both the primary and secondary diagnoses of substance or alcohol related disorders and of personality disorders. 4) The unequal interval between admission and the complete neuropsychiatric assessment reflects the initial uncooperativeness of the hostile and violent cases. However, since the duration of the time frame for assessment was 7 days, the short delay in assessment of these cases is not likely to introduce a significant evaluation bias. This conclusion is also supported by the *worse *scores of many scales in hostile and violent cases despite their assessment after more days of treatment.

### Low prevalence of violent behavior

The present study's results confirm the low rate of patients' violent behavior in Italian PICUs. The findings are simple: yet explaining these findings is anything but simple. One possible explanation is the low admission rate of patients with a primary diagnosis of substance use disorder to Italian PICUs. Actually, Italian Mental Health Departments are not involved in the treatment of patients with a primary diagnosis of substance use disorders. Whether the general features of the Italian society or the specific characteristics of the Italian PICUs (small units with no more than 15 beds located in general hospitals; no special unit for violent patients), or the cultural background of the Italian psychiatric reform could account for the low rates of inpatients' violent behavior is uncertain and difficult to assess.

### Modality of hospitalization

In accordance with previous studies [22,23], greater length of hospital stay was observed in hostile in comparison with non-hostile cases and in violent in comparison with hostile cases, owing to more severe psychopathology or a greater reluctance of clinicians to discharge recently violent patients. Involuntary cases were over-represented in the hostile and even more in the violent group. Aggressive or violent behavior may be both a cause and a consequence of commitment.

### Risk Factors

Clinical rather than socio-demographic variables (with the notable exception of young age) appear more related to the risk of violence. This finding has practical importance because clinical symptoms are amenable to therapeutic approaches. Despite extensive research and speculation about gender as possible risk factor of violent behavior in psychiatric patients, the results of previous studies are inconsistent. Some researchers have reported that males with schizophrenia commit severe acts of violence more frequently than females [24-26], others reported that less severe aggression is more frequent among women with schizophrenia than among men with the disorder [27,28], whereas most authors have found no gender differences in aggression among patients with schizophrenia [5,29-34]. In the present study, sex, years of education, and social class were not related with hostility or violence. Risk factors specifically associated with violence were current younger age, suicidal risk, and diagnosis of schizophrenia. Risk factors associated with hostile and violent behavior were younger age at the onset of the disorder, being single, having no children, lower current and last year best GAF scores, higher BPRS hostility, SAPS, and CGI scores, lower BPRS anxiety-depression score, higher antipsychotic, benzodiazepines, and VPA doses, more frequent use of typical neuroleptics, diagnosis of mania, personality disorder, substance and alcohol related disorders, no diagnosis of depression. Some of these factors are in part tautologically related to hostile and violent behavior, e.g., lower current GAF score (aggressive behavior is an important criterion to give a patient a low GAF score), higher BPRS hostility, SAPS scores (some items of SAPS refer to hostility and aggressive behavior), while others (e.g., higher doses of psychoactive drugs) reflect the need to manage hostility and violence. The finding of higher BPRS hostility and SAPS scores in hostile and violent cases is consistent with the results of Arango et al [2] who found that violent inpatients presented more severe positive psychotic symptoms (suspiciousness, hostility, hallucinations, thought disorder) and poorer insight into delusions and control of aggressive impulses compared with non violent inpatients. Younger age at the onset of the disorder, being single, and having no children are factors associated also with the severity of mental illness in general. Therefore, it is not surprising to find them associated with the most ominous facets of mental disorders. Consistently, last year best GAF score, index of global severity level of the illness, was lower in hostile than in non-hostile cases and lower in violent than in hostile cases. In accordance with previous studies [35-37], the present results show that specific factors related to hostility and violence are the diagnoses of personality disorder, substance or alcohol related disorder, and mania. On the other hand, the diagnosis of depression resulted associated with no hostility. Curiously, SANS score was lower in the hostile group than in the violent and in the non-hostile groups. This was the only result showing the non-hostile and the violent groups similar to each other and different from the hostile group. This apparently paradoxical result might be due to the higher prevalence of depression in the non-hostile group and of schizophrenia in the violent group. Actually, the so called negative symptoms are heterogeneous and include depressive symptoms as well as deficit symptoms of schizophrenia. The factors specifically related with violence deserve special consideration. 1) Younger age has been associated with violent behavior [38]. A similar trend has been observed in psychiatric patients by most [5,7,9,10,30-32,35,39-43], but not all authors [44]. 2) The suicidal risk was found higher in the violent group, although this group was characterized by a low prevalence of diagnoses of depression. This result is consistent with previous studies showing that both suicidal acts and externally directed aggression tend to coexist in the same individual, possibly because of common biological correlates, e.g. decreased serotonergic activity, increased noradrenergic and dopaminergic activity [45]. About 30% of violent individuals have a history of self-destructive acts and, conversely, 10–20% of suicidal individuals have a past history of violent behavior towards others [46]. 3) An increased risk of violence among patients with schizophrenia has been confirmed by evaluation of criminal records [24-26,47], a twin study [44], and studies on psychiatric inpatients [29,30,39,48,49]. While both high levels of hostility and violence were found related with mania, only violence was related with schizophrenia, reflecting a pattern of unexpected, unforeseeable violent behavior specific of the latter disease. Hostility in schizophrenia might be masked by prominent negative symptoms such as flat affect, emotional withdrawal, lack of empathy and it might be unrecognized until comes out as violent behavior.

### Architectonic and staff variables

Despite striking differences in the architectonic characteristics of the buildings where the PICU was located, the rate of hostility and violence resulted similar, suggesting that the architectonic features of the psychiatric unit do not play a major role in influencing patients' aggressive behavior. Similar rates of hostility and violence were observed in the 6 consecutive years considered. In this period, there was a large turn-over of nursing staff but minimal turn-over of psychiatrists. The general attitude of staff members and the style of work [9] did not change.

### Drug treatment

The use of higher antipsychotic, benzodiazepine, and VPA doses in violent than in hostile cases and in hostile cases than in non-hostile cases reflect the physicians' attempt to control patients' risky behavior by increasing drug dosage. The more frequent use of typical neuroleptics in hostile and in violent cases than in non-hostile cases raises the doubt whether the use of neuroleptics is preferred by clinicians to manage patients' hostile and violent behavior or may by itself induce hostility and violence increasing unpleasant feeling in patients, e.g., akathisia, dysphoria, physical discomfort. Both hypotheses are not mutually exclusive, and the explanations for the findings in this study may ultimately derive from a blend of the considerations offered. However, the authors believe that the major factor is the tendency of psychiatrists to trust more in the high doses of neuroleptics than in the recommended dose of atypicals in managing potentially violent patients. The use of higher doses of *all *psychoactive drugs (excluding antidepressants) in the treatment of hostile or violent patients supports our suspicion. Furthermore, akathisia score was similar in the three groups. The higher scores of rigidity and akinesia in non-hostile cases (in spite of the use of lower doses) may be related with the more sensitivity of non-hostile, non agitated cases to antidopaminergic drugs. Akinesia may be also a symptom of depression, a diagnosis more frequent in non-hostile cases.

### PHR

The literature on PHR and seclusion support the following: 1) seclusion and PHR are efficacious in preventing injury and reducing agitation; 2) it is nearly impossible to operate a program for severely symptomatic individuals without some form of seclusion or PHR; 3) demographic and clinical factors have limited influence on rates of PHR and seclusion; 4) training in prediction and prevention of violence, in self-defense, and in implementation of PHR and/or seclusion is valuable in reducing rates and untoward effects; 5) studies comparing well-defined training programs have potential usefulness [50]. In our PICU, the frequency of PHR is low (2,5‰). In other Italian PICUs the use of PHR is much less rare. In American psychiatric units, a mean frequency of 8.5% of PHR has been reported [51]. In an American child and adolescent state psychiatric hospital, a rate of 49% of PHR has been recently reported [52]. Much variance in the use of PHR is probably due to a lack of definite and unifying rules or to non clinical factors like cultural biases, staff role perceptions, and the attitude of the hospital administration [53]. In Italian psychiatric meetings, endless discussions have been made whether PHR is an acceptable form of psychiatric treatment or whether it is preferable to aggressive drug treatment. Although randomized controlled trials are, and will be, unavailable with respect to this issue, the subject should be discussed in the light of empirical data. Looked at from this perspective, the results of the present study indicate that avoiding or minimizing the use of seclusion and PHR does not necessarily results in: 1) more frequent or serious patients' assaults; 2) use of unusually high doses of antipsychotics or other psychoactive drugs; 3) death or high risk of treatment-related side effects.

The evaluation of doses and side effects of psychoactive drugs employed in settings with and without routine PHR could shed light on the possible overuse of drugs in units where PHR is avoided. Likewise, monitoring of the ominous medical consequences of PHR, including death, [54-56] must be implemented to assess the effectiveness and safety of PHR since it is reasonable to assume a considerable number of unreported emergency or fatal cases [57]. In addition, future studies are needed to ascertain patients' point of view, e.g., evaluating the degree of patients' distress remembering involuntary drug treatment or PHR in those who have undergone both treatments. There is urgent need to clarify this issue since the wide range of use of PHR in different psychiatric facilities (from near 0 to several decades for cent) is difficult to be understood and accepted.

### Management of patients after discharge

Patients who present violent behavior in the hospital should receive special attention after discharge, since previous violent behavior is one of the most strong risk factors of further aggressiveness. Adequate preventive strategies should be arranged. In the community-based services, comprehensive treatment with evidence-based biomedical and psychosocial treatment has been found associated with a reduction in the aggressive misconduct of patients with psychotic symptoms [58].
